# Preoperative lymphocyte count, neutrophil to lymphocyte and platelet to lymphocyte ratio predict the recurrence with progression and cancerization in vocal fold lesions—retrospective study

**DOI:** 10.7717/peerj.15642

**Published:** 2023-09-20

**Authors:** Anna Rzepakowska, Wioletta Pietruszewska, Michał Żurek, Maria Molga-Magusiak, Michał Leszczyński, Kazimierz Niemczyk

**Affiliations:** 1 Department of Otorhinolaryngology Head and Neck Surgery, Medical University of Warsaw, Warsaw, Poland; 2 Department of Otiatrics, Laryngology and Laryngological Oncology, Medical University of Łódź, Łódź, Poland; 3 Doctoral School, Medical University of Warsaw, Warsaw, Poland

**Keywords:** Laryngeal cancer, Laryngeal dysplasia, Lymphocyte count, Neutrophil count, Neutrophil to lymphocyte ratio, Blood markers

## Abstract

**Backgrounds:**

This study explored the contribution of peripheral blood markers in diagnosis and prognosis estimation of different stages of laryngeal dysplasia and early glottic cancer.

**Methods:**

Retrospective analysis of clinical, histopathological and laboratory data of 220 patients including hemoglobin, neutrophil, lymphocyte, monocyte and platelet counts, neutrophil to lymphocyte ratio (NLR), platelet to lymphocyte ratio (PLR).

**Results:**

The mean hemoglobin level and platelets count showed differences between histopathological stages of lesions (*p* = 0.041 and 0.046, respectively). In patients with recurrent lesions mean level of lymphocyte count, NLR and PLR were significant in assessing progression and cancerization (*p* = 0.005, 0.028 and 0.023, respectively). The univariate analysis recognized level of PLR ≥ 141.74 as significant risk factor of the recurrence of vocal fold hypertrophic lesions (OR = 1.963).

**Conclusions:**

The levels of blood cells and their ratios seem to be effective in predicting the recurrence of lesion and even more their potential role in indicating malignant progression.

## Introduction

The incidence rates of laryngeal cancer have been steadily decreasing in developed countries over recent decades, however the mortality and morbidity related to this disease is still significant global problem (Cancer Over Time, 2023; Cancer Today, 2023). The invasive squamous cell cancer of larynx is proceeded in over 90% by precancerous changes of laryngeal mucosa (Malzahn et al., 2002); therefore, efforts should focus on early detection of changes and identification of factors that promote malignant transformation. The process of laryngeal carcinogenesis is a multifactorial and still unrevealed thoroughly. Tobacco and alcohol are common and long known etiologic factors of benign, precancerous and malignant laryngeal lesions (Johnson et al., 2020). The model of cancer progression related to the accumulation of DNA changes in epithelial cells, induced by carcinogens, was widely explored in the past (Gaykalova et al., 2014). However the evidence of oncogene overexpression together with the silencing of tumor suppressor genes have not thoroughly explained the stepwise progression from low to high grade dysplasia and development of invasive cancer (Gale et al., 2000; Miyaguchi, Olofsson & Hellquist, 1990). Currently, the influence of the microenvironment on tumor progression is intensively investigated, especially regarding the interaction of host’s immune system and cancer cells (Trivedi, Rosen & Ferris, 2016). The tumor cells induce the inflammatory reaction of the host, which can adversely promote tumor progression by facilitating the angiogenesis, proliferation and metastasis (Coussens & Werb, 2002). The role of tumor-associated neutrophils in promotion of tumor activity by inducing immune tolerance of the stroma is emphasized (Shen et al., 2014; Gregory & Houghton, 2011). However, not only the local immunological markers demonstrated the relationship to outcomes for solid tumors of different sites, but also systemic hematologic markers as neutrophil, lymphocyte, monocyte, and platelet counts proved significance in the prognosis (Moon et al., 2016). The most widely investigated and validated indicator of systemic inflammation is neutrophil-to-lymphocyte ratio (NLR) (Templeton et al., 2014). The studies of various malignant tumors have shown that preoperatively elevated NLR correlate with poorer prognosis, including increased risk of recurrence, metastasis and death related to cancer progression (Miyamoto et al., 2018; Xiao et al., 2014; Ozdemir et al., 2014; Yodying et al., 2016; Gu et al., 2016). The relationship between pretreatment NLR and outcomes has been also investigated across different head and neck squamous cell cancer sites, including larynx and has indicated strong association of elevated NLR with decreased survival (Mascarella et al., 2018; Yang et al., 2019; Sheng et al., 2019; Song et al., 2019; Chen et al., 2018). Monocyte counts, platelet counts and platelet-lymphocyte ratio are other hematological parameters of systemic inflammatory response emerged in published data as potential prognostic factors for patients with cancer (Tsai et al., 2014; Racz et al., 2015; Krenn-Pilko et al., 2014).

In contrast, there are only few studies exploring the alterations in serum markers in early stages of tumor progression. The dysplastic lesions do not breach the basal lamina. Therefore, we expect that blood cell counts changes may appear with invasive cancer stage and serve as potential biomarker in differential diagnosis of intraepithelial laryngeal changes. Therefore, the aim of the study was to explore the contribution of peripheral blood markers in diagnosis and prognosis estimation of laryngeal dysplasia comparing to early glottic cancer.

## Materials & Methods

The protocol of the study was approved by the local Bioethics Review Board (approval number AKBE/137/2021) and the study was conducted in accordance with the ethical principles of Declaration of Helsinki.

This retrospective study was performed by searching archived medical records of patients treated for the first time with laryngeal microsurgery due to suspicious hypertrophic lesions of the vocal folds in two tertiary departments. The following inclusion criteria were established: (1) no previous history of laryngeal microsurgery or radiotherapy due to laryngeal pathology; (2) the preoperative endoscopic examination of the larynx revealed hypertrophic or ulcerated lesion located in glottic area and the mobility of vocal folds was normal; (3) the procedure of laryngeal microsurgery was a radical excision of the entire pathology; (4) the histopathological examination revealed keratosis or dyskeratosis of the squamous cell with no dysplasia, low grade dysplasia, high grade dysplasia or invasive cancer; (5) there was available the complete blood count examination with differential, obtained within 2 weeks prior the laryngeal microsurgery; (6) at least 1-year follow up in outpatient clinic was available. We excluded patients with: (1) laryngeal lesions of clinical benign appearance, *e.g.*, polyps, Reinke’s edema, granuloma, cysts; (2) clinical evidence of acute or chronic inflammatory status (elevated body temperature, antibiotic treatment, chronic steroid or non steroid anti-inflammatory therapy); (3) hematologic diseases; (4) autoimmune disorders; (5) history of other malignancy.

**Figure 1:**
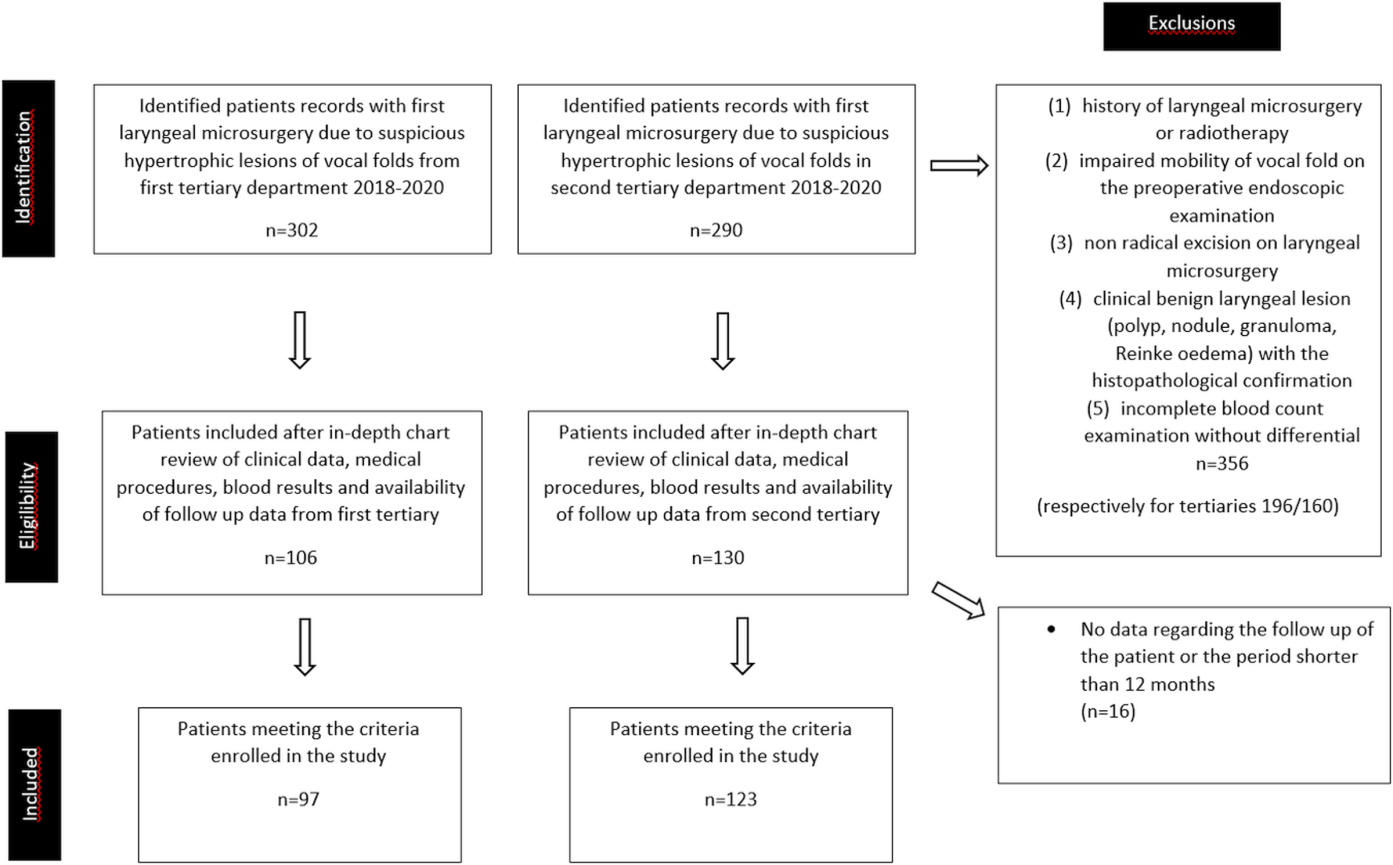
Patient identification, eligibility, and inclusion.

The final analysis was performed on 220 patients from two tertiary hospitals in Poland (Warsaw and Łódź) (Figure 1). We reviewed medical records and extracted data regarding age, sex, preoperative complete blood count with differentiation, protocol of the surgery, histopathology and the outcomes during at last one year observation. The hemoglobin, neutrophil, lymphocyte, monocyte and platelet counts were collected and the ratios were calculated as a division of the absolute counts of: neutrophil to lymphocyte (NRL), platelet to lymphocyte (PLR). After histopathology results were obtained, the patients were divided in four groups according to the 4th edition of the World Health Organization Classification of Head and Neck Tumours from 2017: non dysplastic, low grade dysplasia, high grade dysplasia, invasive cancer (Gale, Poljak & Zidar, 2017). The analysis of outcomes considered the recurrence of the hypertrophic lesions within the glottis among the study group as well as the progression of the intraepithelial lesion’s stage in histopathology. The recurrence was defined as reappearance of the lesion in the same localization of glottis after successful healing of the epithelium post microsurgical resection.

Parameters were evaluated using IBM SPSS Statistics 25.0 (Chicago, IL, USA) and Statistica 13.3. For all analyses a *p*-values were calculated, values < 0.05 were considered to be statistically significant. The features of patients with and without recurrence were compared using Pearson’s chi-squared test and Mann–Whitney *U* test. The blood and biochemical parameters between different groups of patients were evaluated using one-way ANOVA on ranks and Mann–Whitney *U* test. Dunn-Boferroni *post-hoc* tests were used in order to avoid alpha error accumulation.

Using ROC-curves cut-off values of blood and biochemical parameters were identified based on a modified Kolmogorov–Smirnov test. Cut-off value means a value that has the best true positive rate (sensitivity) and the worst false positive rate (1-specificity) to assess the risk of recurrence. For the values odds ratios (OR) with 95% confidence intervals and *p*-values were calculated using univariate logistic regression.

## Results

Among the included 220 patients, men were dominating (148) over women (72). The mean age of the group was 60.06 ± 12.22 (range: 23–93). 69.5% of patients were older than 55 years. According to the histopathology, there were 127 non dysplastic patients, 25 with low grade dysplasia, 22 with high grade dysplasia and 46 with invasive cancer. The recurrences were identified in 64 patients (29.1%) and there was following distribution of unfavorable outcome of the disease among different histopathology stages: 22.8% in non-dysplastic patients, 48% in low grade dysplasia, and 45.5% for high grade dysplasia and 28.3% for invasive cancer. The mean age and sex proportions were comparable for the recurrence and non-recurrence groups. The median of the follow-up was 2,51 years (916 days), range: 0,96–3,85 years. The histopathological status of the recurrence was in the vast majority the same as the primary diagnosis (76.5%). Disease progression but without cancerization was identified in nine (14.1%) cases and cancer invasion in 6 (9.4%). The detailed characteristic of the study group was presented in Table 1.

**Table 1:** Characteristic of patients.

Characteristic	No of patients/years	%		*p*-value
Total (*n*)	220	100		
Sex (*n*)				
Male	148	67.3		
Female	72	32.7		
Mean age; age range (years)	60.06 ± 12.22 (range: 23–93)			
Age at diagnosis (years)				
≥55	153	69.5		
<55	67	30.5		
Histopathological diagnosis (*n*)				
Non-dysplastic	127	57.7		
Low grade dysplasia	25	11.4		
High grade dysplasia	22	10.0		
Invasive cancer	46	20.9		
Disease outcomes (*n*)				
Recurrence	64	29.1		
No recurrence	156	70.9		
Mean age (years)				
Recurrence	60.42 ± 11.91			
No recurrence	59.91 ± 12.38			0.389^a^
Men/women (*n*)				
Recurrence	45/19	70.3/29.7		0.538^b^
No recurrence	103/53	66/34		
Recurrence according to diagnosis (*n*)		% from all lesions:	% from recurrences:	
Non-dysplastic	29	22.8	45.3	
Low grade dysplasia	12	48.0	18.8	0.022^b^
High grade dysplasia	10	45.5	15.6	
Invasive cancer	13	28.3	20.3	
Disease progression or cancerization on recurrence (*n*)				
No progression	49	76.5		
Progression without cancerization	9	14.1		
Cancerization	6	9.4		

**Notes.**

a Mann Whitney *U* Test.

b Pearson’s chi-squared test.

As presented in Table 2, the mean neutrophil, lymphocyte and monocyte count, hemoglobin level as well as NLR and PLR were estimated according to histopathological grade of the lesions, disease outcomes and the status of the recurrence. However, no significant differences were identified for most of the analysed parameters between the groups. The mean platelets count showed significant decrease in low grade dysplasia compared to other stages (*p* = 0.046). The mean hemoglobin levels decreased significantly with the histopathological stage of the lesion (*p* = 0.041). Unfortunately, both results were rejected according to Dunn-Bonferroni *post-hoc* tests. There was also determined decrease in lymphocyte count and increase in NLR and PLR in cases with the recurrence that progressed to invasive cancer (*p* = 0.005, 0.028 and 0.023, respectively), the results were confirmed with Dunn-Bonferroni *post-hoc* tests.

**Table 2:** Mean blood and biochemical parameters (with standard deviations) according to histopathological diagnosis of vocal folds hypertrophic lesions.

Parameter (*n*)	Neutrophil (×10^9^/l)	Lymphocyte (×10^9^/l)	Monocyte (×10^9^/l)	Platelet (×10^9^/l)	Hemoglobin (g/l)	NLR	PLR
Total *n* = 220	4.97 ± 1.76	2.31 ± 0.96	0.67 ± 0.37	243 ± 62.77	14.32 ± 1.38	2.5 ± 1.43	123.33 ±64.75
Histopathological diagnosis							
Non dysplastic *n* = 125	4.94 ± 1.89	2.38 ± 1.09	0.67 ± 0.41	245.19 ± 66.74	14.53 ± 1.4	2.46 ± 1.56	125.84 ±76.86
Low grade dysplasia *n* = 25	4.34 ± 1.03	2.01 ± 0.81	0.59 ± 0.14	213.04 ± 53.68	14.49 ± 1.22	2.54 ± 1.41	115.02 ± 37.22
High grade dysplasia *n* = 22	5.11 ± 1.83	2.24 ± 0.51	0.76 ± 0.35	254.45 ± 37.88	13.98 ± 1.11	2.47 ± 1.22	121.85 ± 43.62
Invasive cancer *n* = 45	5.37 ± 1.63	2.31 ± 0.79	0.69 ± 0.36	247.96 ± 62.62	13.83 ± 1.43	2.6 ± 1.17	121.71 ± 47.02
*p*-value^a^	0.125	0.146	0.32	0.046^*^	0.041^*^	0.533	0.625
Disease outcomes							
Non-recurrence *n* = 153	4.76 ± 1.67	2.19 ± 0.98	0.65 ± 0.28	238.83 ± 68.06	14.17 ± 1.15	2.56 ± 1.48	131.9 ± 79.54
Recurrence *n* = 64	5.07 ± 1.8	2.36 ± 0.95	0.68 ± 0.41	244.75 ± 60.58	14.39 ± 1.47	2.48 ± 1.42	119.75 ±57.37
*p*-value^b^	0.399	0.117	0.361	0.343	0.296	0.753	0.600
Disease progression/cancerization on recurrence							
No progression *n* = 49	4.76 ± 1.62	2.36 ± 1.01	0.63 ± 0.23	233.76 ± 65.28	14.12 ± 1.1	2.37 ± 1.53	114.79 ± 55.73
Progression without cancerization *n* = 9	4.01 ± 1.5	1.36 ± 0.59	0.53 ± 0.08	250.33 ± 100.01	14.6 ± 1.47	3.25 ± 1.15	227.41 ± 137.1
Cancerization *n* = 6	5.87 ± 1.97	2.06 ± 0.44	0.95 ± 0.59	263 ± 15.92	13.85 ± 0.98	3.02 ± 1.22	128.28 ± 23.16
*p*-value^a^	0.146	0.005^*^	0.236	0.181	0.907	0.028^*^	0.023^*^

**Notes.**

a *p*-value according to results of one-way ANOVA on ranks.

b *p*-value according to results of Mann–Whitney *U* test.

* *p* < 0.05.

The performed univariate analysis of factors that correlated with the recurrence of vocal fold hypertrophic lesions recognized the level of PLR ≥ 141.74 (*p* = 0.038, OR = 1.963) statistical significant risk factor. The values of all analyzed factors are presented in Table 3.

**Table 3:** Univariate logistic analysis of factors related to vocal fold lesions recurrence.

	No (%)	OR	95% CI	*P*-value
Age (years)				
≤55	16 (24.2)	1.456	0.753–2.815	0.264
>55	48 (31.8)			
NLR				
<3.84	50 (27.2)	1.975	0.921–4.235	0.08
≥3.84	14 (42.4)			
PLR				
<141.74	41 (25.6)	1.963	1.038–3.713	0.038^*^
≥141.74	23 (40.4)			
Neutrophil (×10^9^/l)				
<4.33	26 (29.2)	1.023	0.565–1.852	0.94
≥4.33	38 (29.7)			
Lymphocyte (×10^9^/l)				
≤3.63	59 (29.1)	1.356	0.436–4.216	0.599
>3.63	5 (35.7)			
Monocyte (×10^9^/l)				
≤1.05	59 (28.6)	2.076	0.610–7.066	0.242
>1.05	5 (45.5)			
Platelet (×10^9^/l)				
≤201.5	13 (25.5)	1.296	0.673–2.639	0.474
>201.5	51 (30.7)			
Hemoglobin (g/l)				
≤13.5	17 (27.4)	1.152	0.599–2.217	0.672
>13.5	47 (30.3)			

**Notes.**

* *p* < 0.005.

## Discussion

NLR as well as PLR are confirmed nonspecific indicators of oncological outcomes of different site tumors, including head and neck squamous cell cancers. NLR is of particular importance and there are even suggestions to incorporate it into the prognostic systems for some cancers to help predict resistance, unfavorable prognosis and tailor individual therapy (Galizia et al., 2015; Cantiello et al., 2018).

The ongoing debate concerns the nonspecificity of the markers and their relationship with many other conditions, *e.g.*, infection, inflammatory diseases, cardiovascular diseases and medicines (Bae et al., 2020). However the opponents cite an example of lactate dehydrogenase, other nonspecific parameter, that is widely used prognostic marker for non-Hodgkin’s lymphomas. The mechanisms explaining the connection between NLR and PLR and oncologic outcomes are still not thoroughly revealed. The recognized contribution of neutrophils in tumorigenesis involves the angiogenesis promotion and cell growth induction by secreting hepatocyte growth factor, vascular endothelial growth factor (VEGF) and matrix metalloproteinase (Xiao et al., 2014; Coffelt, Wellenstein & De Visser, 2016). Additionally, the subset of neutrophils, that produce proteases, reactive oxygen species and upregulate programmed death-ligand 1 (PD-L1), demonstrate also the suppression of anti-tumor activity of the cytotoxic lymphocytes, natural killer cells, and activated T cells (Oberg et al., 2019). Simultaneously, tumor cells activate platelets in the microenvironment, which in turn release proangiogenic VEGF supporting tumor survival and proliferation. Activated platelets also release transforming growth factor *β*1, that promotes epithelial-mesenchymal transition (EMT) and increase tumor cells motility, facilitating invasion and metastasis (Sharma et al., 2014).

So far a number of studies have been published presenting association between neutrophil and plates counts and oncological outcomes in laryngeal cancer, but there are only a few studies analysing those parameters in laryngeal dysplastic lesions (Shen et al., 2014; Templeton et al., 2014; Mascarella et al., 2018; Yang et al., 2019). The studies investigating the microenvironment reactivity on progression of laryngeal dysplasia and malignant transformation are also limited (Trivedi, Rosen & Ferris, 2016). The exploration of immune cells contribution to host response in the process of intraepithelial changes progression and initiation of laryngeal cancer invasion would aid the field and in the future help to stratify patients and individualize the therapy. The research by Kum et al. (2014) was the first investigating the NLR in the benign and precancerous laryngeal lesions with comparison to advanced stages of laryngeal cancer. They proved significant differences of mean NLR between those groups, however failed to find differences between mild, moderate and severe dysplasia subgroups (Kum et al., 2014). Kara et al. (2019) conducted quite similar study and presented significant differences of NLR and PLR values between benign, precancerous laryngeal lesions and T1 glottic cancer; however the examination within the precancerous changes revealed no significant difference of ratios between subgroups of mild, moderate and severe dysplasia.

The recent study by Fang et al. (2019) analysed large sample of vocal fold leukoplakia and correlated their histopathology and postoperative outcomes with blood markers. However, when they analysed histopathology groups according to the earlier WHO classification of intraepithelial laryngeal lesions with six stages (no dysplasia, mild, moderate, severe, carcinoma *in situ*, invasive cancer), the differences of NLR and PLR were not evident. Only the adaptation of a two-tier risk classification system (low and high risk lesions) and a subgroup comparison revealed significant difference of both NLR and PLR (Fang et al., 2019). Moreover, they confirmed significantly higher rates of NLR and PLR in patients with disease relapse compared to those with no recurrence, and the differences were even higher for cases with cancer transformation (Fang et al., 2020).

The results of our study also affirm that the blood parameters are not effective in determining the severity of laryngeal dysplasia. However, their role in terms of prognosis is of interest in regard to further analysis, especially considering wide availability and low costs of blood tests.

A major advantage of the study is the double-checking of the statistical significance of the results, not only with the *p*-value determined by statistical tests, but also with *post-hoc* tests. This strengthens the reliability of the results obtained and the project from other published studies.

The retrospective design is the major limitations of our study. The potential prognostic value of mentioned hematological parameters regarding recurrence or progression of laryngeal dysplasia requires prospective, multicenter, randomized controlled studies to comprehensively evaluate the findings. The follow-up duration of 12 months seems to be quite short for the recurrence or cancerization observation; however the majority of the T1 glottic cancer relapses appear within first 24 months and are asymptomatic (Pakkanen et al., 2021) Moreover, the late relapses are more dependent on continued exposure to risk factors. The early ones are more dependent on internal biological mechanisms. Identification of the factors predictive of the early recurrence is clinically important to verify the treatment options.

It would be most appropriate to combine these studies with histochemical assessment of immune status within tissue samples of laryngeal intraepithelial lesions and their surrounding stroma.

## Conclusions

The levels of blood cells and their ratios do not seem to be effective in determining the stage of laryngeal hypertrophic lesions; however, their effectiveness in prognosing the recurrence of changes and, even more, their potential role in indicating malignant progression is promising.

##  Supplemental Information

10.7717/peerj.15642/supp-1 Data S1 Anonymized database of participants with clinical features and values of blood markers

## Additional Information and Declarations

### Data Availability

The following information was supplied regarding data availability: The raw measurements are available in the Supplementary File.
